# SARS-CoV-2 Phylogenetic Analysis, Lazio Region, Italy, February–March 2020

**DOI:** 10.3201/eid2608.201525

**Published:** 2020-08

**Authors:** Barbara Bartolini, Martina Rueca, Cesare Ernesto Maria Gruber, Francesco Messina, Fabrizio Carletti, Emanuela Giombini, Eleonora Lalle, Licia Bordi, Giulia Matusali, Francesca Colavita, Concetta Castilletti, Francesco Vairo, Giuseppe Ippolito, Maria Rosaria Capobianchi, Antonino Di Caro

**Affiliations:** National Institute for Infectious Diseases “Lazzaro Spallanzani” IRCCS, Rome, Italy

**Keywords:** SARS-CoV-2, Italy, full genome sequence, phylogenetic tree, coronavirus disease, respiratory infections, viruses, severe acute respiratory syndrome coronavirus 2, COVID-19

## Abstract

We report phylogenetic and mutational analysis of severe acute respiratory syndrome coronavirus 2 virus strains from the Lazio region of Italy and provide information about the dynamics of virus spread. Data suggest effective containment of clade V strains, but subsequently, multiple waves of clade G strains were circulating widely in Europe.

Severe acute respiratory syndrome coronavirus 2 (SARS-CoV-2) has raised serious concerns because of its rapid dissemination worldwide. Italy is one of the countries with the highest number of coronavirus disease (COVID-19) cases (*1*,*2*). Nevertheless, the information about the molecular epidemiology of SARS-CoV-2 strains circulating in Italy is still limited. The analysis of sequence data shown in GISAID (https://www.gisaid.org) indicates that the initial introduction of SARS-CoV-2 in Italy through 2 infected tourists in January was effectively contained (*3*), and no further circulation of similar clade V strains has been so far detected. An intense wave of infections occurred afterwards, initially affecting Lombardy and Veneto and later on all the other regions of Italy. The strains detected in Italy since February 20 belonged only to clade G. This clade, apparently originating in Shanghai, has been widely circulating in the European Union (EU) countries before reaching Italy (*3*–*5*).

Preliminary data suggested that multiple introductions of clade G strains have occurred in Italy, giving rise to contemporary circulation of different strains also detected in other EU countries; this pattern suggests that, after partially undetected introduction of the virus in EU from China, the circulation of travelers within EU ignited virus spread in Europe. We report the phylogenetic and mutational analysis of SARS-CoV-2 strains detected in the Lazio region of Italy, providing additional information on the dynamics of virus dissemination in this country.

## The Study

We analyzed nasopharyngeal swab (n = 6) and bronchoalveolar lavage (n = 3) samples from 9 patients with COVID-19 to perform SARS-CoV-2 whole-genome reconstruction and mutational analysis. We collected samples in late February and early March, 2020 (Table 1). At sampling time, all patients reported symptoms such as fever, sore throat, cough, or other respiratory symptoms. Two sequences were identical, so we included only 1 of them in the analysis, resulting in 8 total sequences. We named the sequences INMI3–INMI10 for their detection at National Institute for Infectious Diseases and analyzed them together with the previously published INMI1 and INMI2 (*6*), along with all the sequences from Italy posted to GISAID database by April 11, 2020. 

**Table 1:** Demographic and epidemiologic data for patients with severe acute respiratory syndrome coronavirus 2, Italy, 2020

Characteristic	INMI3	INMI4	INMI4bis	INMI5	INMI6	INMI7	INMI8	INMI9	INMI10
Sample type*	NPS	NPS	NPS	NPS	NPS	BAL	NPS	BAL	BAL
Sex	M	M	F	M	M	F	F	M	M
Age, y	32	41	38	53	60	70	65	33	56
Region	Emilia Romagna	Lombardy	Lombardy	Lazio	Lazio	Lazio	Lazio	Lazio	Lazio
Collection date	Mar 1	Feb 28	Feb 27	Mar 4	Mar 23	Mar 23	Mar 7	Mar 23	Mar 4

*BAL, bronchoalveolar lavage; NPS, nasopharyngeal swab.

We performed next-generation sequencing (SARS-CoV-2 Panel) on Ion Torrent platform (Thermo Fisher Scientific, https://www.thermofisher.com) using shotgun approach for INMI3–4 and amplicon approach for INMI5–10. After quality control, we generated a median number of 4.3 × 10^7^ reads for each shotgun sample and 1.5 × 10^6^ for each amplicon sample (ranging from 7.5 × 10^5^ to 4.8 × 10^7^). The sequencing mean depth of SARS-CoV-2 ranged from 367-fold in INMI3 to 16,661-fold in INMI5.

We submitted consensus sequences to GISAID. We used the proposed phylogenetic lineage classification (A. Rambaut et al., unpub. data, https://doi.org/10.1101/2020.04.17.046086) in phylogenetic analysis; for comparison to previously published reports, we maintained references to clades reported in GISAID. INMI1 and INMI2 are included in clade V according to GISAID phylogenetics, as reported (*6*), and clade B2; the clade includes other sequences from EU countries, but no additional sequences from Italy. All other INMI sequences cluster with the GISAID G clade, and with the B1 clade; we focused subsequent analysis on clade B1 (Figure).

**Figure:**
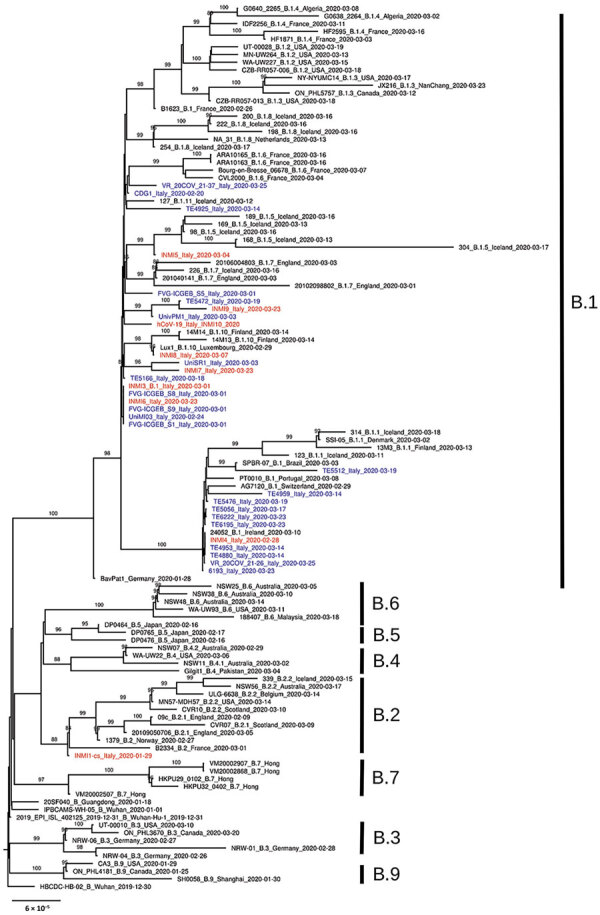
Phylogenetic analysis of 150 severe acute respiratory syndrome coronavirus 2 representative genome sequences, including genomes collected in Italy (blue) and sequences identified for this study at the National Institute for Infectious Diseases (red). Available genomes were retrieved from GISAID (https://www.gisaid.org) on April 10, 2020; we discarded sequences with low coverage depth (low amount of read sequenced) or low coverage length (not complete genome sequences). Representative sequences from every B lineage (A. Rambaut et al., unpub. data, https://doi.org/10.1101/2020.04.17.046086v1), together with all genome sequences collected in Italy so far, were selected for further analysis. Multiple sequence alignment was obtained with MAFFT version 7.271 (https://mafft.cbrc.jp/alignment/software). Phylogenetic analysis was performed with IQ-TREE (http://www.iqtree.org): transition model with empirical base frequencies and invariable sites was selected with ModelFinder, and the best tree was found performing 1,000 bootstrap ultrafast replicates. Bootstrap values of >80% are reported on each branch. Lineages according to the description by Rambaut et al. are marked to the right of the tree. Scale bar represents number of substitutions per site.

The clade B1 INMI sequences are distributed in 2 main clusters, one including most of the northern Italy strains and the other including sequences mainly from central Italy. In particular, INMI4, which was epidemiologically linked to Bergamo (Lombardy region), clusters with sequences from central Italy (Abruzzo region). The other INMI sequences cluster with strains from northern Italy. Of note, in both clusters the sequences from Italy are intermixed with sequences from other EU countries, which can also be seen in the broader phylogenetic analysis on GISAID, in which more EU sequences are analyzed. We have identified 5 synonymous and 9 nonsynonymous substitutions distributed along the whole genome (Table 2).

**Table 2:** Consensus sequences of severe acute respiratory syndrome coronavirus 2 samples, Italy, 2020*

Wuhan-Hu-1 strain	Nucleotide position	INMI3	INMI4	INMI5	INMI6	INMI7	INMI9	INMI8	INMI10	Amino acid	Region
A	187					G				Noncoding	UTR
C	241	T	T	T	T	T	T	T	T	Noncoding	UTR
C	2062								T	Noncoding	UTR
C	3037	T	T	T	T	T	T	T	T	Syn	Orf1ab
G	4255						T			Syn	Orf1ab
C	14408	T	T	T	T	T	T	T	T	P4715L	Orf1ab
T	16456						G			S5398A	Orf1ab
A	20268			G						Syn	Orf1ab
C	21575					T				L5F	Spike glycoprotein
A	23403	G	G	G	G	G	G	G	G	D614G	Spike glycoprotein
C	23575							T		Syn	Spike glycoprotein
A	26530						G			D3G	Membrane glycoprotein
C	28881		A							R203K	Nucleocapsid protein
G	28882		A							R203K	Nucleocapsid protein
G	28883		C							G204R	Nucleocapsid protein
*Nucleotide positions refer to the Wuhan-Hu-1 reference genome (GenBank accession no. MN908947). Orf, open reading frame; Syn, synonymous substitution; UTR, untranslated region.											

Each patient showed several amino acid substitutions ranging from 4 to 7. The G clade–specific single-nucleotide polymorphism A23403G led the amino acid change D614G in the S protein. We observed one additional mutation in this protein, that of C21575T (L5F) in INMI7, which is detected in few other sequences in GISAID, interspersed among different non-G clades (M. Chiara et al., unpub. data, https://doi.org/10.1101/2020.03.30.016790). Its location in a marginal region of the gene and the sporadic distribution in different clades indicates repeated occurrence not followed by fixation, consistent with no evolutionary advantage.

The S protein in the SARS-CoV-2 virus is a chief determinant of the host range and pathogenicity. The virion attaches to the cell membrane by binding the S protein with the host ACE2 receptor (*7*). The D614G mutation, located in the putative S1–S2 junction region near the furin polybasic cleavage site (RRAR), might have an effect on priming by host cell proteases; however, the real impact of this high-frequency mutation is unclear.

The variants C241T, C3037T (located in the noncoding region) and C14408T (in open reading frame1ab, orf1ab) were present in all INMI3–INMI10 sequences. These mutations have been detected in several SARS-CoV-2 isolates throughout Europe and are characteristic of clade G (C. Yin, unpub. data). A nonsynonymous substitution D3G in membrane glycoprotein was detected in 1 INMI9 sequence.

We detected 3 nucleotide changes in INMI4, located in a high variable region of the gene, in 2 adjacent codons of the nucleocapsid (N) gene, two 2-amino acid changes, R203K and G204R. N protein, responsible for the formation of helical nucleocapsid, can elicit humoral and cell mediated immune response and has potential value in vaccine development. However, none of the observed mutations has been so far associated with changes in viral pathogenicity or transmissibility.

## Conclusions

The phylogenetic reconstruction we report suggests possible multiple introduction of SARS-CoV-2 virus in Italy, supporting previously reported analysis conducted on a more limited number of sequences (*3*–*5*).

The analysis consistently places the strains described in this study in 2 distinct clusters in B1 clade. No other sequence from Italy clusters in B2 (or GISAID V) clade, indicating the positive effect of containment measures established by health authorities in both Italy and China to limit viral transmission directly from China. The same measures were unable to contain a wave of subsequent multiple introductions in Italy of strains that were widely circulating in Europe, all clustering with clade B1.

The inclusion of the viral sequences from infections occurring in the Lazio region helps to demonstrate the dynamics of virus circulation in Italy. In particular, a small number of mutations have been detected in these strains, but the real impact and role that these mutations may have on the pathogenicity and transmissibility of SARS-CoV-2 remains to be determined.

A limitation of our research is that only a portion of viral sequences, including the sequences from Italy, have been published as of April 10, 2020; phylogenetic analysis could substantially change when more sequences are made available. Continued genomic surveillance strategies are needed to improve monitoring and understanding of current SARS-CoV-2 epidemics, which might help to lessen the public health impact of COVID-19. Furthermore, increased sequencing capacity is necessary for contact tracing and enhanced surveillance activity.
